# Specific gene module pair-based target identification and drug discovery

**DOI:** 10.3389/fphar.2022.1089217

**Published:** 2023-01-16

**Authors:** Peng Li, Chujie Bai, Lingmin Zhan, Haoran Zhang, Yuanyuan Zhang, Wuxia Zhang, Yingdong Wang, Jinzhong Zhao

**Affiliations:** ^1^ Shanxi key lab for modernization of TCVM, College of Basic Sciences, Shanxi Agricultural University, Jinzhong, Shanxi, China; ^2^ Department of Orthopedic Oncology, Peking University Cancer Hospital & Institute, Key Laboratory of Carcinogenesis and Translational Research (Ministry of Education), Beijing, China

**Keywords:** transcriptome, gene module pair, drug target prediction, drug discovery, drug–target association

## Abstract

Identification of the biological targets of a compound is of paramount importance for the exploration of the mechanism of action of drugs and for the development of novel drugs. A concept of the Connectivity Map (CMap) was previously proposed to connect genes, drugs, and disease states based on the common gene-expression signatures. For a new query compound, the CMap-based method can infer its potential targets by searching similar drugs with known targets (reference drugs) and measuring the similarities into their specific transcriptional responses between the query compound and those reference drugs. However, the available methods are often inefficient due to the requirement of the reference drugs as a medium to link the query agent and targets. Here, we developed a general procedure to extract target-induced consensus gene modules from the transcriptional profiles induced by the treatment of perturbagens of a target. A specific transcriptional gene module pair (GMP) was automatically identified for each target and could be used as a direct target signature. Based on the GMPs, we built the target network and identified some target gene clusters with similar biological mechanisms. Moreover, a gene module pair-based target identification (GMPTI) approach was proposed to predict novel compound–target interactions. Using this method, we have discovered novel inhibitors for three PI3K pathway proteins PI3Kα/β/δ, including PU-H71, alvespimycin, reversine, astemizole, raloxifene HCl, and tamoxifen.

## Introduction

When the sequencing of the human genome identifies risk-associated loci or genetic mutation for diseases, understanding the biological function and effects of the corresponding genes (proteins) is the top priority in the life science study. Similarly, for drugs with unknown molecular mechanisms, identification of their mechanistic targets is of paramount importance for the development of novel drugs. Truly understanding the biological effects of drugs requires monitoring the molecular pathways targeted by drugs and the subsequent impacts, such as the overall gene expression profiles. Evidently, omics techniques are naturally suited for capturing these systemic effects, such as transcriptomics, proteomics, and metabonomics (Trapotsi and Hosseini-Gerami, 2022). Until now, there have been many large-scale databases that integrate different types of omics data induced by genetic or compound perturbation on biological samples (Barrett et al., 2013; Sjöstedt and Zhong, 2020; Wishart et al., 2022). Among them, the low-cost transcriptomics is the most useful for detecting functional associations between drugs and genes, as the constructed compendia of comprehensive and uniform-quality genetic and compound-induced gene expression data, such as the Connectivity Map (CMap) (Lamb et al., 2006; Subramanian et al., 2017). The CMap-based concept is a potential solution and has established systematic, large-scale compendia of the cellular effects of pharmacological and genetic perturbation. CMap-based approaches explore the actions of compounds by comparing their induced gene-expression profiles with the gene-expression profiles of perturbagens with known mechanisms. For example, if a query compound has expression profiles similar with the landmarked compounds with known mechanisms of action or genetic perturbagens, we can infer the compound has similar upstream targets or pathways with the landmarked compounds and genetic perturbagens (Qu and Rajpal, 2012; Musa et al., 2018).

Until now, two versions of CMap have been built. The pilot (old) CMap database contains 6,100 gene-expression profiles obtained by the treatment of a set of 1,309 different molecules (Lamb et al., 2006). Since then, CMap-based methods have been widely used for discovering the drug’s mode of action and drug repositioning. For example, based on CMap, Brum et al. found that parbendazole can induce osteogenic differentiation and explored withaferin A, calcium folinate, and amylocaine as potential osteogenic drugs (Brum et al., 2015; Brum et al., 2018). Manzotti et al. (2015) found that amantadine is associated with monocyte–macrophage-like differentiation of myeloid leukemia cell lines. Liu et al. (2015) explored celastrol as a sensitization of leptin, and it can be used to treat obesity. In recent years, in view of the small scale of the pilot CMap dataset, the L1000 platform expands the CMap resource in different dimensions including the number of perturbations, cell lines, doses, and times (Subramanian et al., 2017). The new version CMap can further accelerate the discovery of drug actions. For example, Chen et al. (2021) used the L1000 platform to discover hyperforin as a stimulator of thermogenesis by stimulating AMPK and PGC-1a *via* a Ucp1-dependent pathway. van Leeuwen et al. (2022) integrated the L1000 data and identified drugs that potentiate the anti-breast cancer activity of statins. In addition, the large-scale transcriptomic data of genetic and chemical perturbations from the CMap database also provide opportunities for updating current computational pharmacogenomics and drug design methodologies. For example, Zhang and Gant (2008) proposed a novel pattern matching the algorithm named statistically significant connectivity map (ssCMap) to help reduce noise effects in CMap-based approaches. Fortney et al. (2015) presented a method, CMapBatch, which adapted parallelly processed multiple-gene signatures. The L1000CDS^2^ search engine optimized CMap data and methods to improve the ability of knowledge extraction from the CMap platform (Duan et al., 2016).

The CMap-based methods explored connections among drugs, pathways, and diseases by measuring the gene-expression signature similarity. However, the perturbagens as a medium are indispensable for these efforts to discover the biological connections. If we want to predict a potential drug–target interaction, the query drug has to be linked to targets mediated by perturbagens in the CMap database. Because of the diversity of treatment conditions, the same perturbagens might connect to the query drug with sharply different scores and make users hard to determine which one is suitable. To solve this problem, we developed a general procedure to capture target-induced consensus gene modules hidden in the transcriptional profiles following the treatment of target’s perturbagens across multiple cell lines and dosages. A specific transcriptional gene module pair (GMP) was automatically extracted for each target and can be used as a gene signature to represent the target. Based on the GMPs of targets, we built the target network by calculating the similarity among GMPs of all targets and identified some target gene clusters with similar biological mechanisms. Moreover, the gene module pair-based target identification (GMPTI) approach was proposed to predict novel compound–target interactions based on a compound-induced gene expression profile.

## Materials and methods

### Data source and preprocessing

All LINCS-funded CMap L1000 data are available from GEO. Both LINCS Phase 1 data in GEO Series GSE92742 and LINCS Phase 2 data in GEO series GSE70138 were combined. The L1000 platform carries out a rigorous five-step data-processing pipeline to transform raw data from Luminex scanners to replicate consensus signatures. The final LEVEL 5 data were used in this work. It totally contains 594,697 signatures (118,050 in GSE70138 and 473,647 in GSE92742). The L1000 assay directly measures 978 landmark genes and infers additional 11,350 genes. Of the inferred genes, 9,196 are well inferred. Our work only used the high-fidelity 10,174 genes, including 978 measured landmarks and 9,196 well-inferred genes.

We collated gene targets for all perturbagens from the cloud-based computing environment termed CLUE (Connectivity Map Linked User Environment), available at https://clue.io/. Genetic perturbagens refer to two types of knockdown (KD) or overexpression (OE) on targeted genes. The effects of compounds on targets were artificially annotated. These perturbagens with clear targets were then mapped to the LEVEL 5 data to extract corresponding signatures. As a result, 138,310 signatures for 5,852 perturbagens with 4,540 gene targets were retained for this study.

### Distance between two signatures

The distance between two signatures was measured by a modified gene set enrichment analysis (GSEA)-based method (Iorio et al., 2010). Given two signatures *X* and *Y*, following the work of Iorio et al., we selected 250 upregulated genes $up=\left\{g_{1},\cdots ,g_{250}\right\}$ and downregulated genes $dn=\left\{g_{1},\cdots ,g_{250}\right\}$ to represent each signature. The distance between two signatures was defined as follows:

$$d_{X,Y}=\frac{{ITES}_{X,Y}+{ITES}_{Y,X}}{2},$$

where

$${ITES}_{X,Y}=1-\frac{abs\left({ES}_{Y}^{X_{up}}-{ES}_{Y}^{X_{dn}}\right)}{2}.$$

Here, $I{TES}_{X,Y}$ , defining the distance from *X* to *Y*, is the inverse total enrichment score of the signature *X* gene sets {*up*, *dn*}, with respect to the signature of *Y*. ${ES}_{Y}^{X_{r}}$ (with *r* ∈ {*up*, *dn*}) is the enrichment score of the signature of *X* (the upregulated part and the downregulated one) with respect to the signature of *Y*. Similarly, ${ITES}_{Y,X}$ describes the distance from *Y* to *X*.

$${ITES}_{Y,X}=1-\frac{abs\left({ES}_{X}^{Y_{up}}-{ES}_{X}^{Y_{dn}}\right)}{2}.$$

Then, we performed a hierarchical cluster analysis for all signatures using the calculated distances.

### Cluster analysis of signatures for each target

For a target, its signatures denote all signatures of perturbagens of this target. We clustered signatures for each target on their pairwise distance values and plot the dendrogram. Then, signatures cut by a pre-defined threshold of 0.8 in the dendrogram were considered outliers and removed in the dendrogram of each target.

The distance threshold value (i.e., 0.8) was determined by the following considerations. First, a significant threshold was estimated by a multiple random sampling approach. In all 138,310 signatures, we randomly selected 1,000 signatures and calculated pairwise distances between them, resulting in $\left(\begin{array}{l} 1000\\ 2 \end{array}\right)$ = 499,500 distance values. The empirical probability distribution function (pdf) of these data was used to estimate a significance threshold for the distance. The upper bound of the 5% quantile of this empirical pdf was chosen as the distance significance threshold value. This procedure was repeated 1,000 times, and the mean of 1,000 threshold values approximately 0.8 was retained as the significant threshold. Based on the calculated threshold value, we manually inspected each cluster tree of the 4,540 targets and selected 0.8 as the threshold to remove outliers. Finally, 4,461 were retained with at least three signatures.

### Co-expression analysis

It was hypothesized that on-target gene expression effects of different perturbagens for the same target should be similar and co-expressed. To find co-expression module genes induced by one target, we performed a co-expression analysis for signatures of each target using the weighted correlation network analysis (WGCNA) method (Langfelder and Horvath, 2008).

### Target-specific gene modules

After the co-expression analysis, those genes that were not in any co-expressed modules were removed from signatures of each target. To extract the target-specific gene modules from co-expressed genes, the Borda merging method implementing a majority voting system was used to sort genes according to their values in each signature:

$$G=\left[g_{1},g_{2},g_{3},{\cdots ,g}_{n},\right],$$

$$v_{g_{i}}=\overset{m}{\underset{j=1}{\sum }}v_{g_{i}}^{j},$$

where *G* is a ranked gene list of size *n* by sorting the corresponding merging value $v_{g_{i}}$ for each gene $g_{i}$ , in decreasing order. $v_{g_{i}}$ denotes the sum (merging value) of $v_{g_{i}}^{j}$ in signatures 1 to *m*. $v_{g_{i}}^{j}$ is the value of gene $g_{i}$ in signatures *j*.

To this step, each target corresponds to a gene list *G*, among which specific gene modules for this target can be extracted. We selected the top 250 genes (*t*_*up*_) of each gene list and the bottom 250 ones (*t*_*down*_) as the target-specific gene module pair (*t*_*up*_, *t*_*down*_).

### Characterization of the target-specific gene module pair in human gene networks

InWeb_Inbiomap (Inbiomap) focuses on a scored physical protein–protein interactions (Li et al., 2017), available from https://www.lagelab.org/resources/. Pathway commons (Pathcom) was downloaded from http://www.pathwaycommons.org/. Pathcom concentrates on biological pathways integrated from public pathway and gene interactions (Rodchenkov et al., 2020). The Search Tool for Recurring Instances of Neighboring Genes (STRING; https://string-db.org) quantitatively integrates different studies and interaction types into a single integrated score for each gene pair based on the total weight of evidence (Snel et al., 2000). The Genome-scale Integrated Analysis of gene Networks in Tissues (GIANT; https://hb.flatironinstitute.org/) network covers functional association genes and inferred functional relations (Huang et al., 2018).

We analyzed the enrichment of the module gene members in the network by calculating the ratio of protein–protein connections among the fully connected network. When both top and bottom modules were analyzed together, the fully connected network has $\left(\begin{array}{l} 500\\ 2 \end{array}\right)=124,750$ links. When the top and bottom modules were analyzed, the fully connected network has $\left(\begin{array}{l} 250\\ 2 \end{array}\right)=31,125$ links. The significance of the enrichment was measured by comparing the actual ratio with that of a random model. In the random model, a collection of genes with the same number as the module genes was randomly selected from the network, and then the connection ratio was calculated. This step was repeated 1,000 times, and a null distribution was constructed.

### Target network

The similarity between two targets is estimated by the number of intersection genes between the two targets’ specific module pairs. The more overlapping the genes are, the more similar the two targets are. Then, we considered each target as a node in the network and connected two nodes with a weighted edge, if their similarity is below a significant threshold value. To evaluate the significance of the linkage between targets, we generated a null distribution for each target by randomly permuting top and bottom transcriptional modules and repeated the calculation 1,000 times for target connections. This null model uses the gene module-based permutation test procedure and preserves gene–gene correlations of the gene expression data, providing a more biologically reasonable assessment of significance than would be obtained by permuting genes. The edge weight is proportional to the similarity that is intersection genes of two targets’ specific module pairs, where the significant threshold is computed by the hypergeometric test (*p* < 0.05).

### Target community identification

The affinity propagation algorithm is used to identify target communities in the target network (Frey and Dueck, 2007; Bodenhofer et al., 2011). This algorithm takes in the target pairwise similarity matrix and outputs a set of clusters. Each cluster is represented by a cluster center data point called exemplar, whose features best interpolate the features of all the other points in the cluster.

### Specific gene module pair-based target identification

GMPTI considers experiments with gene-expression profiles from a collection of samples belonging to two classes, for example, drug treated vs. control. The genes can be ordered in a ranked list *L*, according to their differential expression between the classes. Given the defined GMP for each target, the goal of GMPTI is to compare *L* to each target-specific GMP using a similarity metric slightly adjusted with that used in gene set enrichment analysis (Subramanian et al., 2005). We defined the raw similarity score as follows:

$${{TCS}_{L}^{t}=ES}_{L}^{up}-{ES}_{L}^{down},$$

where ${ES}_{L}^{up}$ is the enrichment of *t*_*up*_ for *L*, and ${ES}_{L}^{down}$ is the enrichment of *t*_*down*_ for *L*. ${TCS}_{L}^{t}$ denotes the total correlation score of the GMP (*t*_*up*_, *t*_*down*_) of one target, with respect to signature *L*. The total correlation score (*TCS*) ranges between −2 and 2. It measures the degree of similarity between query *L* and target-induced gene-expression profiles. It will be positive for targets that are positively related to *L*, negative for those that are inversely similar, and near zero for signatures that are unrelated. A zero value is assigned when both ${ES}_{L}^{up}$ and ${ES}_{L}^{down}$ are the same sign.

### Normalization of similarity scores

To allow for the comparison of similarity scores across multiple expression datasets, the scores are normalized to account for differences in query ranked gene lists. GMPTI normalizes the ${TCS}_{L}^{t}$ values within each ranked gene list as follows:

$${NCS}_{L}^{t}=\frac{{TCS}_{L}^{t}}{\mu },$$

where ${NCS}_{L}^{t}$ and *μ* are, respectively, the normalized correlation score and the absolute mean of ${TCS}_{L}^{t}$ (the mean of absolute values) for all target-specific module pairs corresponding to the query gene list. By normalizing TCS , GMPTI accounts for differences in correlations between GMPs and the expression dataset; therefore, the normalized correlation scores (*NCS*) can be used to compare the analysis results across different expression profiles.

### Estimating significance

We assess the significance of an actual NCS value by comparing it with the set of scores NCS_NULL_ computed with random permutations of both top and bottom gene modules for each target. 1) We generated a random GMP for each target by randomly permuting top and bottom transcriptional modules in our target space. 2) Step 1 was repeated for 1,000 permutations, and a histogram of the corresponding similarity scores NCS_NULL_ was created for a query gene list. 3) A nominal *p*-value for the NCS*i* of a target *i* was estimated by using the portion of the NCS_NULL_ distribution above the actual NCS*i* as follows:

$$P=\frac{N(abs({NCS}_{NULL)}\geq abs({NCS}_{i})}{1000},$$

where $abs({NCS}_{NULL}$ is the absolute value of all correlation scores for random GMPs with respect to a query gene list *L*. $abs\left({NCS}_{i}\right)$ is the absolute value of the similarity score of target *i* with respect to *L*.

### PI3Kα/β/δ kinase assay

The test compounds including varenicline tartrate, PU-H71, alvespimycin, reversine, astemizole, raloxifene HCl, and tamoxifen were purchased from Shanghai Aladdin Biochemical Technology Co., Ltd. PI3Kα/β/δ were purchased from Carna Biosciences. This study aims to determine the effect of test compounds on PI3Kα/β/δ enzyme activity using ADP-Glo-based biochemical assay (Vendor: Promega, Cat#: V9102), following the manufacturer’s instruction. The classical PI3K inhibitor wortmannin was used as a positive control. Luminescence signal (RLU) is detected for each well by using a multimode plate reader (Vendor: BioTek, Cat#: Synergy4) and converted to % inhibition. Then, the IC50s were calculated by fitting % inhibition values and the log of compound concentrations to the hill slope with the variable slope (called the variable slope model or four-parameter dose-response curve), and the log (inhibitor) vs. response curve was built by GraphPad Prism version 7.0 (GraphPad Software). Data are presented as mean ± SEM, with n = 3 for each drug dose.

## Results

### Target-specific gene module pair

It was hypothesized that on-target gene-expression effects of different perturbagens for the same target should be similar and co-expressed. For a gene target, its specific GMP indicates two gene sets that are specifically expressed at the top and bottom of the gene-expression profiles induced by perturbing this target. To extract the GMP for each target, we exploited a library of gene transcriptional responses to different perturbagens (e.g., small-molecule compounds and shRNAs): the newly expanded Connectivity Map (CMap) containing 476,251 gene expression profiles (consolidating replicates) obtained by the treatment of 77 different human cell lines at different dosages with a set of 27,927 perturbagens (Subramanian et al., 2017). We collected gene targets of all perturbagens from CLUE. Then, each target was mapped to its transcriptional signatures that are the differential gene profiles induced by the perturbagens of the target including both small-molecule compounds and shRNAs. As a result, 138,310 signatures for 5,852 perturbagens with 4,540 gene targets were retained.

**FIGURE 1:**
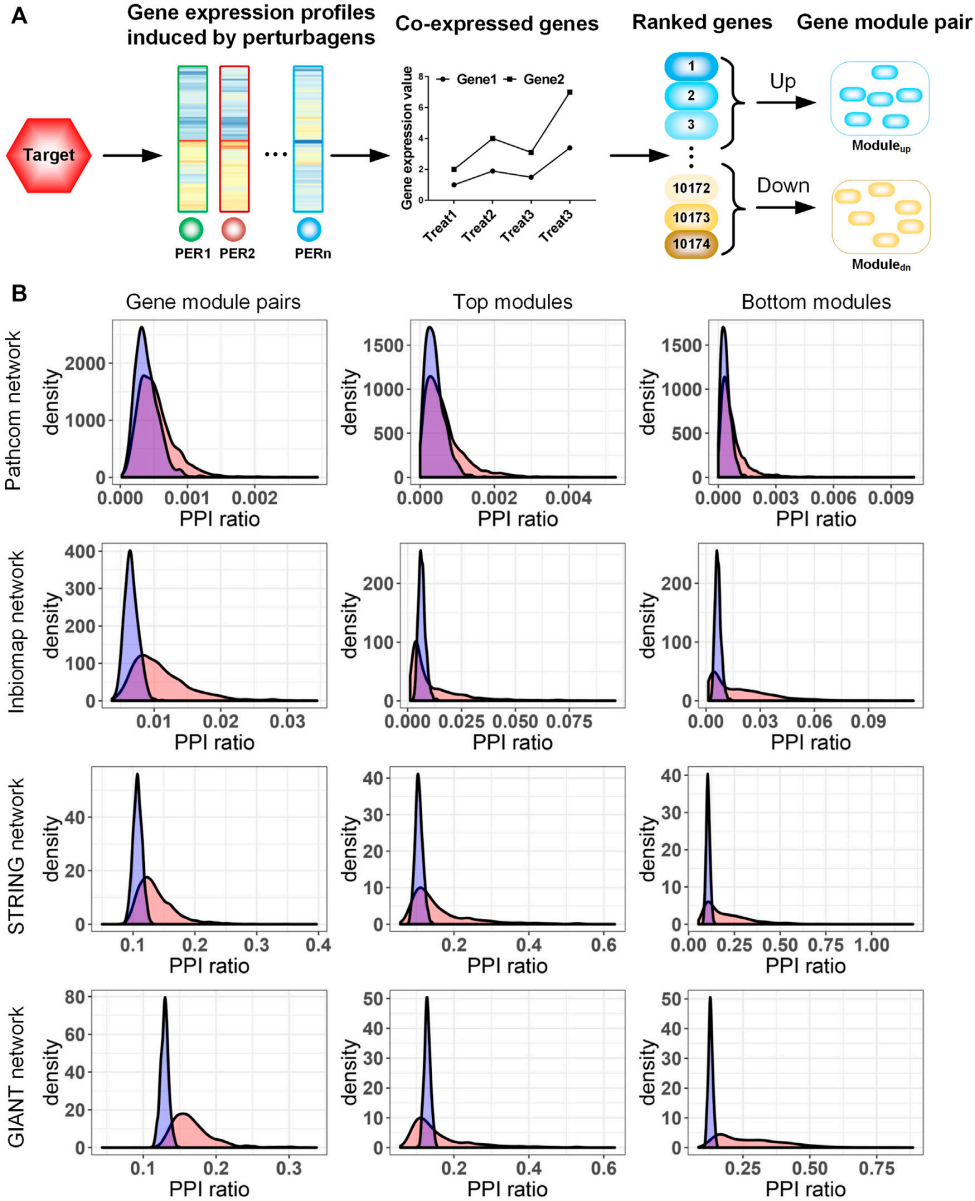
**(A)** Procedure to extract the gene module pair (GMP) of a target. **(B)** Characterization of the target-specific GMP in human gene networks. Four types of gene networks were collected from Pathcom, Inbiomap, STRING, and GIANT. We evaluated the functional enrichment of module genes in each network by calculating the ratio of protein–protein interaction numbers among the link numbers of fully connected networks (PPI ratio). The actual PPI ratio is compared with that of a random model to assess the significance of the enrichment. In the random model, a collection of genes with the same number as the module genes were randomly selected from the network, and then the PPI ratio was calculated. The distribution with blue and red colors is random and observed data, respectively. Rows 1–4 rows correspond to the analysis of the Pathcom, Inbiomap, STRING, and GIANT networks, respectively. Columns 1–3 correspond to analysis of gene module pairs, top modules, and bottom modules, respectively.

Based on these data, we proposed a novel method to extract the GMP for a target (Figure 1A). First, we integrated co-expression genes for each target by performing the WGCNA on its signatures. For a target’s signatures, there may be some outliers that are distinct from most signatures and are difficult to reflect transcriptional activities induced by perturbing this target. To reduce the influence of these outliers in the construction of GMPs, we clustered signatures for each target on their pairwise distances and removed outlier signatures in the dendrogram by a pre-defined threshold (see Materials and Methods). The distance between two signatures was measured by a modified GSEA-based method (Iorio et al., 2010). In order to equally weight the contribution of all signatures to the co-expressed genes, the Borda merging method, implementing a majority voting system, was used to sort the co-expressed genes according to their ranks in each signature. The GMP including the two top/bottom gene sets was extracted from the merged gene list by selecting the first 250 genes at the top of the gene list (most overexpressed) and the last 250 ones at the bottom of the gene list (most downregulated) following a previous work (Iorio et al., 2010). Finally, the GMPs were successfully constructed for 3,505 targets. Out of these, we noted that the GMPs for 229 targets were integrated from only a small number of multi-target perturbagens. For example, the GMP of adiponectin receptor protein 2 (ADIPOR2) was concluded by 70 signatures of the compound parthenolide, which is not only an adiponectin receptor agonist but also an NF-κB inhibitor. For these targets, it is hard to judge the specificity of their GMPs; thus, they were removed from the target space. The existing 3,275 targets were confidential, and their GMPs capture the consensus transcriptional response of the targets across different perturbations, reducing non-relevant effects due to off-target, dosage, or cell line (Supplementary Table S1).

### Characterization of the target-specific gene module pair in human gene networks

To check the functional coherence of target-induced transcriptional modules, we compared their gene members in four genome-wide interaction networks with different gene interaction types. Out of networks, Inbiomap focuses on a scored physical protein–protein interactions (Li et al., 2017). Pathcom concentrates on biological pathways integrated from public pathway and gene interactions (Rodchenkov et al., 2020). STRING quantitatively integrates different studies and interaction types into a single integrated score for each gene pair based on the total weight of evidence (Snel et al., 2000). The GIANT network covers functional association genes and inferred functional relations (Greene et al., 2015). These networks differing in both interaction type and coverage (Supplementary Table S2) could systemically evaluate the function relation of the target-induced gene modules in this study.

We first analyzed the gene members of both top/bottom modules together. In the four networks, we observed that Pathcom enriched a minimum of 758 (∼22%) GMPs compared with its null model (nominal *p*-value <0.05; Figure 1B), though this number is evidently less than that in other networks. The three networks, Inbiomap, STRING, and GIANT, significantly cover more gene relations than their corresponding null models on at least 2,200 GMPs (49%), while 1,180 GMPs (26%) were enriched in all the three networks (nominal *p*-value <0.05; Figure 1B). Moreover, the top and bottom modules were analyzed. In agreement with functional analyses of the combined co-expression modules, except Pathcom, all networks enriched a large amount of modules (from 1,000 to 1,981 upregulated modules and from 1,000 to 2,331 downregulated modules) (nominal *p*-value <0.05; Figure 1B). These results indicate that gene members in target-induced transcriptional modules are mostly functionally relevant and cover a diversity of molecular interaction types.

### Gene module pair based-target gene map

**FIGURE 2:**
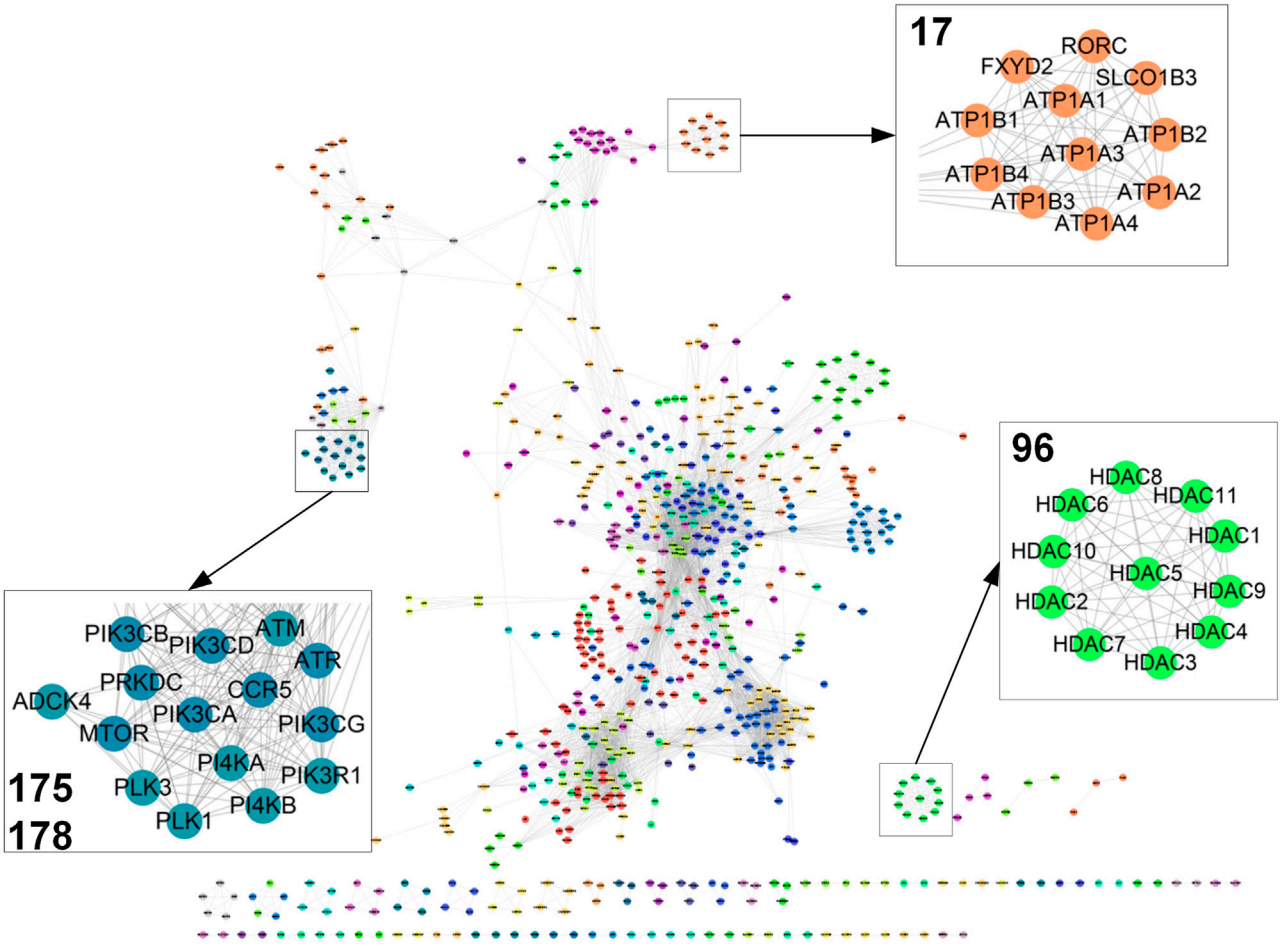
Target network. Part of the target map with 5,000 links were displayed (see more details in Supplementary Table S4). Clusters 17, 96, 175, and 178 were magnified (see more details in Supplementary Table S5).

GMPs reflecting the transcriptional response of targets’ perturbation can be used to relate different target genes. The similarity between collections of GMPs allowed us to calculate a target map connecting target genes together through sequential linkage. The similarity was estimated by the quantity of intersections between two GMPs. Then, we consider each target as a node in the network and connected two nodes with a weighted edge, if their similarity is below a significant threshold value. To evaluate the significance of the linkage between targets, we generated a null distribution for each target by randomly permuting top and bottom transcriptional modules and repeated the calculation 1,000 times for target connections. This null model uses the gene module-based permutation test procedure and preserves gene–gene correlations of the gene expression data, providing a more biologically reasonable assessment of significance than would be obtained by permuting genes (See methods). It can be seen that 2,593 (∼82.5%) targets are connected in a map with 221,275 edges (permutation based *p*-value < 0.05; Figure 2A; Supplementary Table S3), corresponding to ∼4% of a fully connected network with all 3,275 targets (5,361,175 edges).

To further detect the target relations, the affinity propagation algorithm is used to identify target clusters in the target map. This algorithm takes in the target pairwise similarity matrix and outputs a set of clusters. Each cluster is represented by a cluster center data point called exemplar, whose features best interpret the features of all the other points in the cluster. We identified 225 clusters with at least two target nodes in the target map (Figure 2A; Supplementary Table S4). Each cluster was coded with a numerical identifier. As only gene expression information is used to calculate the cross-target similarity, each cluster should reflect a similar transcriptional regulatory activity of biologically related targets. As expected, we observed that targets with similar functions cluster together in the map. For example, 11 histone deacetylases gather in Cluster **96**. Likewise, sodium/potassium-transporting ATPase proteins stay together in Cluster **17**. Also, target genes within a pathway should co-localize and intra-connect in the map as their similar transcriptional regulatory activity. Thus, PI3 and PI4 kinase sets localize with other kinases including ATM, ATR, PLK1, PLK3, and MTOR.

### Gene module pair-based target identification

**FIGURE 3:**
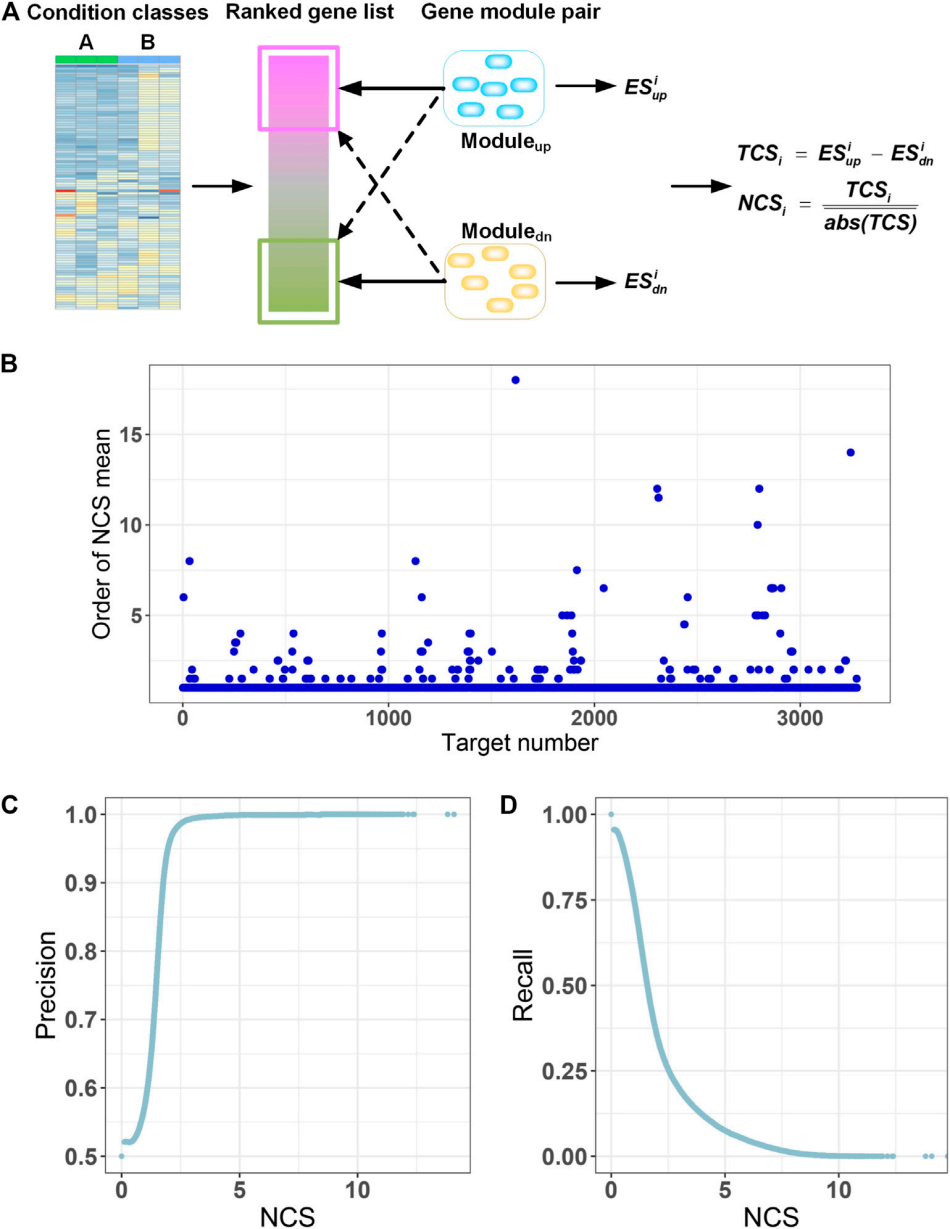
Gene module pair-based target identification**. (A)** A ranked gene list between two classes is compared with GMPs of all targets. A total correlation score (*TCS*_*i*_) is used to quantify the correlation between the gene list and each GMP by an adjusted gene set enrichment approach. Then, the TCS is divided by absolute means of the TCS scores for all GMPs to get a normalized correlation score (*NCS*_*i*_) with respect to the query. **(B)** Mean of NCS for all transcriptional signatures of each target is calculated for all GMPs and ranked. Because there may be multiple transcriptional signatures for a target in the L1000 database, we calculated the mean of multiple NCS values for each target relative to all GMPs. Then, for each target, NCS mean values for all GMPs were ranked in descending order by the absolute values, and the order of the GMP the target itself is extracted and displayed. The horizontal axis displays the 3,275 targets. The vertical axis is the order of the NCS mean value for a target and the GMP of the target itself. **(C)** Precision at different NCS cutoffs. **(D)** Recall at different NCS cutoffs.

GMPTI considers experiments with gene-expression profiles from a collection of samples belonging to two classes, for example, drug-treated vs. control cells. The agent-induced gene expression profiles can be ordered in a ranked list, according to some metrics (e.g., the differential expression values between the two classes). Given the defined GMPs, the goal of our strategy is to compare the correlation of the query gene list with the GMPs of targets (Figure 3A). A strong correlation indicates a similar transcriptional response induced by the agent and the target. The TCS is measured by a method adjusted from that used in the GSEA (see Methods). A positive TCS indicated a similar transcriptional response induced by the agent and the target, and a negative TCS indicated a reversed transcriptional response induced by the agent and the target. To allow for the comparison of scores across multiple queries, we normalized them by dividing a query’s score into absolute means of the raw scores for all GMPs and calculated an NCS with respect to the query. The significance of the normalized score was assessed by comparing it with a null distribution of scores computed by random permutations of top and bottom transcriptional modules in all target spaces.

In addition, we manually tidied the effects of perturbagens for each target including both inhibition and activation that signifies the GMPs were concluded from the transcriptional profiles of inhibitors and agonists of targets, respectively. Out of the 3,275 targets, we found that 3,119 (95.2%) were inhibited, 26 (0.8%) were stimulated, and 131 were undetermined. From these data, we could determine how the query agent induces the corresponding gene-expressional profile in GMPTI. For example, when the transcriptional profile induced by an agent strongly positively correlated with the GMP of a target labeled “inhibited” in the target space, we speculated the agent might induce its gene expression by inhibiting the mechanism related to the target.

The quality of GMPs for each target is of paramount importance for prediction of targets by GMPTI. To assess the quality of GMPs, we used L1000 data as queries to examine whether the GMP of a target can be enriched into the transcriptional signatures of the target itself more than other GMPs. This means the transcriptional signatures of the target will have a greater NCS on its GMPs than other GMPs. We observed that signatures of 3,137 targets (∼95.8%) have maximal NCS on its corresponding GMPs. The signatures of other 138 targets and their own GMPs display slightly lower NCSs ranked from 2nd to 18th in all NCSs (Figure 3B; Supplementary Table S1). Manual inspection of these 138 targets indicated that GMPs with NCSs larger than their own GMPs are mostly corresponding to those targets that have similar biological mechanisms to their own targets. These results indicate that the GMPs of most targets are more correlated with transcriptional signatures of their own or other targets with similar biological mechanisms, confirming the quality of GMPs.

When a drug-induced gene expression profile is known, GMPTI can quantify the functional associations between the drugs and targets with GMPs by using NCS values and the corresponding nominal *p* values. For a drug–target association, the NCS absolute value measures the extent of functional association between the drug and target. The larger the NCS absolute value, the stronger the drug–target functional association. The nominal *p*-value <0.05 means that more than 95% NCS values from the random model are less than the real NCS. We can find drug–target associations by both *P* and NCS values. Generally, a nominal *p*-value <0.05 can be regarded as the minimum standard for filtering potential drug–target associations, which can be further refined by the ranked NCS values. To examine the influence of NCS on the prediction, we regarded NCS as cutoff values and monitored the distribution of the positive predictive value (precision) and true positive rate (recall). As shown in Figures 3C,D, when raising the NCS values, the precision values sharply increase to the maximum, and correspondingly, the recall values gradually decrease, indicating the NCS values can be used to filter drug–target associations.

### Discovery of novel targets of drugs

**FIGURE 4:**
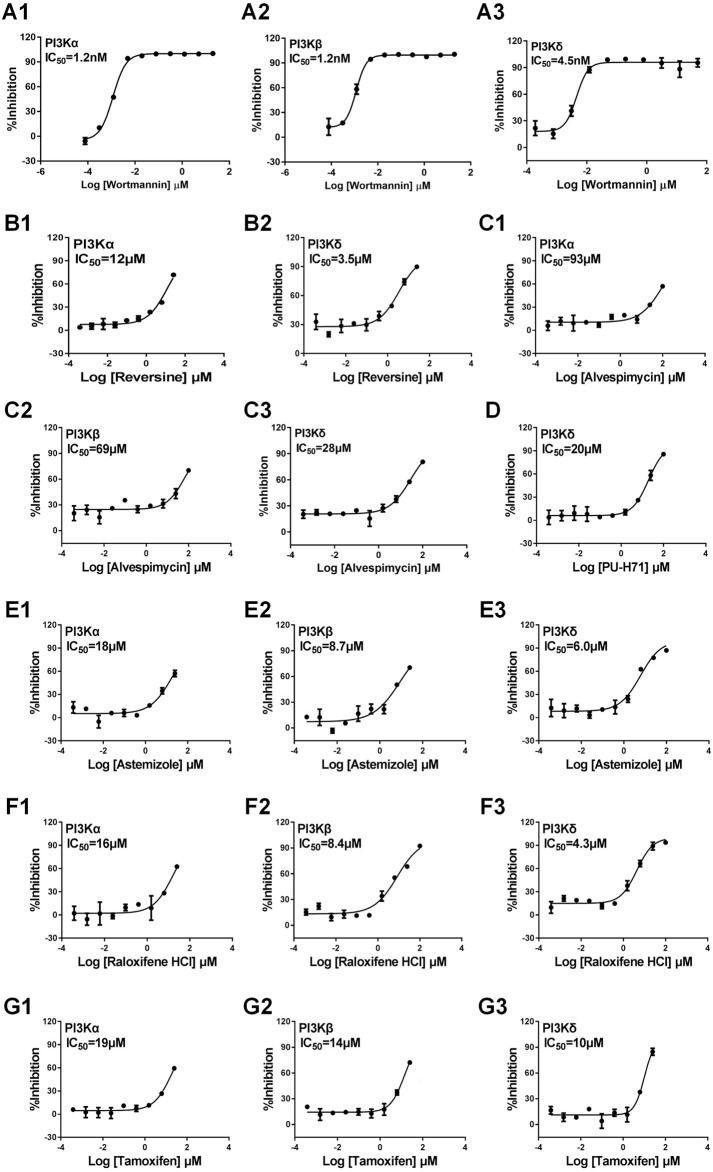
Experimental validation of interaction between the test compound and PI3Kα/β/δ. We used the classical PI3K inhibitor wortmannin as the positive control. **(A1–3)**: Wortmannin and PI3Kα/β/δ; **(B1–2)**: Reversine and PI3Kα/δ; **(C1–3)**: Alvespimycin and PI3Kα/β/δ; **(D)**: PU-H71 and PI3Kδ; **(E1–3)**: Astemizole and PI3Kα/β/δ; **(F1–3)**: Raloxifene HCL and PI3Kα/β/δ; **(G1–3)**: Tamoxifen and PI3Kα/β/δ. Data are presented as mean ± SEM, with n = 3 for each drug dose.

We focused on identifying ligands that act on the PI3K signaling pathway, a key biological process involved in cancer and inflammatory diseases by GMPTI. This pathway has three target genes PI3Kα/β/δ in the target space and is suitable to be taken as an example of this test. First, GMPTI was used to screen 5,520 small-molecular compounds from the L1000 dataset. For each target, we assessed whether it connected to the 5,520 compounds. When these compounds were listed in descending order by NCS values, it was observed that most known ligands for the three targets were top ranked with significant scores. For PI3Kα, 308 compounds exhibited the expected interaction, and out of all 15 known PI3Kα ligands in the L1000 dataset, 14 ligands such as LY-294002, wortmannin, and NVP-BEZ235 were included in the top ranks (nominal *p*-value <0.05, Supplementary Table S6). Similarly, 351 compounds exhibited the expected interaction for PI3Kβ, and out of all 13 known PI3Kβ ligands in the L1000 dataset, 11 ligands were included in the top ranks (nominal *p*-value <0.05, Supplementary Table S6). For PI3Kδ, 321 compounds exhibited the expected interaction, and out of all 14 known PI3Kδ ligands in the L1000 dataset, 13 ligands were included in the top ranks (nominal *p*-value <0.05, Supplementary Table S6). Based on the NCS for the three kinases, we selected three potential compounds, PU-H71, alvespimycin, and reversine, to measure their affinity by direct-binding assay. In this test, we used the classical PI3K inhibitor wortmannin as a positive control, and the results showed that wortmannin inhibits PI3Kα, PI3Kβ, and PI3Kδ with IC50 of 1.2 nM, 1.2 nM, and 4.5 nM, respectively, confirming the specifications of the binding assay (Figures 4A1-A3). Reversine has been known as a novel class of ATP-competitive Aurora kinase (Aurora A, Aurora B, and Aurora C) inhibitor and induces cell cycle arrest and apoptosis. GMPTI showed that reversine might also be a potential PI3K pathway inhibitor with IC50 of 12 µM and 3.5 µM for PI3Kα and PI3Kδ, respectively (Figures 4B1, B2). For alvespimycin and PU-H71, it has been known that both compounds are potent heat shock protein 90 (HSP90) inhibitors. Our model demonstrated that they also have potential to inhibit the PI3K pathway. Among them, alvespimycin slightly inhibited PI3Kα, PI3Kβ, and PI3Kδ with IC50 of 93 µM, 69 µM and 28 µM, respectively (Figures 4C1–C3). PU-H71 has IC50 of 20 µM to antagonize the activation of PI3Kδ (Figure 4D).

The aforementioned prediction is based on the L1000 dataset and might improve the prediction ability of GMPTI. To further test the validity of GMPTI for predicting novel compound–target interactions on external data, we collected the old version CMap dataset that includes 1,309 compounds and their induced gene expression profiles. For each compound, its signature was created by differential genes and was used as a query for GMPTI. For a better comparison, we also predicted the activity of these compounds against the PI3K pathway. GMPTI predicted 410, 374, and 408 compounds were significantly related to PI3Kα, PI3Kβ, and PI3Kδ, respectively (nominal *p*-value <0.05, Supplementary Table S7). In the result, we can see the top three predicted compounds, LY294002, sirolimus, and wortmannin, are known PI3K inhibitors. In addition, we experimentally tested three well-known drugs, astemizole, raloxifene HCl, and tamoxifen, that were repositioned to PI3K inhibitors by GMPTI. Astemizole is known as a second-generation H1-receptor antagonist for use in relieving allergy symptoms, including rhinitis and conjunctivitis. The binding assay confirmed that astemizole inhibits PI3Kα, PI3Kβ, and PI3Kδ with IC50 of 18 µM, 8.7 µM, and 6 µM, respectively (Figures 4E1–E3). Raloxifene is a selective estrogen receptor modulator and is indicated for the treatment of osteoporosis in postmenopausal women and corticosteroid-induced bone loss. We here verified its inhibitory effect on PI3Kα, PI3Kβ, and PI3Kδ with IC50 of 16 µM, 8.4 µM, and 4.3 µM, respectively (Figures 4F1–F3). Tamoxifen, a well-known competitive inhibitor for the estrogen receptor, has been used to treat estrogen receptor-positive metastatic breast cancer. It was also found to be a PI3K inhibitor with IC50 of 19 µM, 14 µM, and 10 µM for PI3Kα, PI3Kβ, and PI3Kδ, respectively (Figures 4G1–G3).

## Discussion

Discovery of molecular mechanisms targeted by a compound is a top priority for the development and application of novel drugs. Direct prediction based on the chemical structure information of drugs usually finds a large number of redundant targets that are unrelated to the pharmacological effects of drugs. CMap-based methods explored connections among drugs, pathways, and diseases using a large collection of transcriptional responses following compound treatments (Lamb, 2007). The L1000 platform expands the CMap resource in different dimensions including the number of perturbations, cell lines, doses, and times (Subramanian et al., 2017). However, the perturbagens as a medium are indispensable for the CMap methods to discover the biological connections. This makes the exploration of the drugs’ mode of action of fuzzy and sometimes need more empirical judgment. We developed a general procedure to capture target-induced consensus gene modules hidden in the transcriptional profiles following the treatment of the target’s perturbagens across multiple cell lines and dosages. Finally, a specific transcriptional GMP was automatically extracted for each target and can be used as a gene signature to represent the target. Based on the GMPs of targets, we built the target network by calculating the similarity among GMPs of all targets and identified some target gene clusters with similar biological mechanisms.

Our approach has the ability to infer mechanisms of queries with known gene-expression profiles. Three proteins PI3Kα/β/δ in the PI3K pathway were taken as an example. We found novel ligands of the three proteins not only in L1000 compounds but also the external dataset. We have experimentally validated three potential compounds PU-H71, alvespimycin, and reversine in the L1000 dataset and three well-known drugs astemizole, raloxifene HCl, and tamoxifen in the old CMap dataset by the direct-binding assay. It should be noted that these drug–target interactions have affinities in the micromolar range in the experimental test and should be aspecific effects. However, the analysis of the binding efficiencies of natural products and marketed drugs indicates that therapeutic efficacy is not necessarily associated with high binding affinity (Mestres and Gregori-Puigjané, 2009). For instance, memantine, a drug for Alzheimer’s disease, is an uncompetitive, low-affinity (in the micromolar range), non-selective N-methyl-D-aspartic acid (NMDA) receptor antagonist, and has less side effects than high-affinity (nanomolar or higher) drugs (Lipton, 2007). In addition, drugs to interact with multiple targets might also have changed to improve efficiency (Hopkins et al., 2006; Ohlson, 2008).

The major limitation of our approach is the limited quantity and quality of perturbagens for a target. The key of our approach is concentrating on the commonalities reserved in the transcriptional responses of different perturbagens for the same target. If the number of perturbagens is too small to cover the most transcriptional features of the target, the extracted GMPs were hardly sufficient to represent the target. The L1000 platform made it possible as the comprehensive, large-scale compendium of functional perturbations of the gene expression resource at various conditions. Certainly, it should be noted that the expression of most genes was not directly measured but inferred in the L1000 assay, although the reliability of the inferred transcripts were theoretically confirmed (Subramanian et al., 2017). In addition, we should note that it is inevitable for a target having perturbagens with inconsistent effects on different situations (for example, different cell lines, doses, and times); merging gene expression profiles from distinct perturbagens might dilute the biological effects of the target. For example, it is well-known that gene expression is drastically affected by drug dosages. The extraction of GMPs from the LINCS level 5 data without considering the impact of dosages could cause dose-dependent biases. Nevertheless, our approach makes a unique identifier for each target by merging profiles from multiple conditions, which give the opportunity to directly build links between targets, drugs, and diseases from a gene transcriptional level.

## Data Availability

The datasets presented in this study can be found in online repositories. The names of the repository/repositories and accession number(s) can be found in the article/Supplementary Material.
